# Dietary Vitamin E Is More Effective than Omega-3 and Omega-6 Fatty Acid for Improving The Kinematic Characteristics of Rat Sperm 

**DOI:** 10.22074/cellj.2016.4322

**Published:** 2016-05-30

**Authors:** AliReza Alizadeh, Zeinab Taleb, Bita Ebrahimi, Vahid Esmaeili, Abdolhossein Shaverdi, Javad Nasr, Abolfazl Kheimeh, Reza Salman Yazdi

**Affiliations:** 1 Department of Animal Science, Saveh Branch, Islamic Azad University, Saveh, Iran; 2 Department of Embryology, Reproductive Biomedicine Research Center, Royan Institute for Reproductive Biomedicine, ACECR, Tehran, Iran; 3 Animal Core Facility, Reproductive Biomedicine Research Center, Royan Institute for Biotechnology, ACECR, Tehran, Iran; 4 Department of Andrology, Reproductive Biomedicine Research Center, Royan Institute for Reproductive Biomedicine, ACECR, Tehran, Ira

**Keywords:** Vitamin E, Fish Oil, Sunflower Oil, Rat, Sperm

## Abstract

**Objective:**

Although key roles for dietary vitamin E (VITE) and fatty acid (FA) in fertility have been confirmed, limited data are available on the effects of VITE alone, or a constant level of VITE supplemented by dietary omega-6 and omega-3 FAs in combination on male reproduction. Consequently in this paper, the effects of VITE, sunflower oil, fish oil and their combination on rat sperm were investigated.

**Materials and Methods:**

We divided 50 mature male Wistar rats into 5 groups (n=10) in a experimental completely randomized design for eight weeks: i. Control (CTR): standard diet; ii. Vitamin E diet (VITE): 2 times greater than recommendations; iii. Sunflower oil group (n-6) [gavaged with 0.5 ml/day/rat sunflower oil+VITE diet]; iv. Fish oil group (n-3): [gavaged with 0.5 ml/day/rat fish oil+VITE diet] and v. n-3+n-6 group [gavaged with 0.3 ml fish oil/day/rat+0.2 ml sunflower oil/day/rat+VITE diet]. The sperm parameters were measured by computer assisted semen analyzer (CASA). All data were analyzed with SPSS software.

**Results:**

Feed intake decreased in groups which were administered sunflower oil compared with the other groups (P<0.05). The groups which received only VITE or fish oil+VITE had a significantly higher concentration of sperm compared with the n-6+n-3 and CTR group (P<0.05). VITE and n-3 showed significant improved progressive motility compared to the CTR group, whereas the n-6 and n-6+n-3 groups were in the middle (P<0.05). The highest sperm kinematic parameters were observed in the VITE only group. There was no strong correlation between sperm parameters and blood lipid profiles.

**Conclusion:**

Dietary VITE and fish oil+VITE can improve sperm quality. Our findings can be a focus for improvements in sperm quantity and motility in fertile animals using only dietary VITE.

## Introduction

Although key roles for vitamin E (VITE) and sperm fatty acid (FA) profiles in fertility have been confirmed (1,2), limited data are available on the effects of VITE only or a constant level of VITE supplemented by dietary omega-6 and omega-3 FAs as well as the combination of these essential FA on male reproduction. Some omega-3 and omega-6 polyunsaturated fatty acids (PUFA), especially 22-C chain FA such as docosahexaenoic acid (DHA, C22:6 n-3) for humans and ruminants; docosapantaenoic acid (DPA, C22:5 n-6) for boars, rodents and rabbits; and docosatetraenoic acid (DTA, C22:4 n-6) for domestic birds are recognized as major components in spermatozoa. Membranes with a high content of 22-C chain PUFA in their phospholipids are distinguished by high levels of flexibility, compressibility, deformability, and elasticity (3). On the other hand, high levels of PUFA increase the susceptibility of the sperm cells to free radical induced peroxidative damages, considered a significant cause of male infertility. Within the sperm antioxidant system VITE is the major natural lipid-soluble antioxidant present in cell membranes and plays a crucial role in breaking the chain reaction of peroxidation, initiated by reactive oxygen species (ROS) (4). However, dietary antioxidant support has been neglected in some previous studies, although the study of antioxidant supplementation helps to elucidate the importance of this antioxidant activity when the diet is supplemented by PUFA (3). 

Dietary FAs may influence FA profiles in several organs (5,6). The direct and/or indirect presence of dietary omega-3 PUFA in sperm has been shown to be effective in some species and to improve sperm parameters (7,11). Although manipulation of sperm FA by dietary FA is typical of all experiments, randomized controlled trials have produced conflicting results regarding the improvement of semen parameters with or without VITE supplementation. PUFA supplementation has been shown to disturb sperm parameters in the rodent, *Calomys laucha*, when used without VITE supplementation (12). 

The dietary ratio of omega fatty acids (n-3: n-6) are another concern. Increased consumption of soybean oil, as a source of omega-6, has probably decreased tissue concentrations of Eicosapentaenoic acid (EPA) and DHA during the 20^th^ century in the United States (13). However, little is known about the effects of the combination of omega-6 FA and fish oil on semen quality. 

Thus, the main objective of the present study is to determine the effects of VITE only or VITE in combination with sunflower oil (omega-6 source), fish oil (omega-3 source) and a combination of sunflower and fish oil on rat sperm parameters using a computer-assisted sperm analyzer (CASA), feed intake, and blood lipid profiles. This study also investigates the correlation between blood lipid profiles and sperm quality. 

## Materials and Methods

### Animals and diets

This study was approved by the Ethics Committee of the Royan Institute and follows the Nutrient Requirements of Laboratory Animals (NRC, 1995). In this experiment, 50 male Wistar rats aged 8 weeks that weighed 194 ± 19 g were used in a experimental completely randomized design. The rats were divided into five equal groups. Rats were allocated to treatment groups according to their weight (high, medium and low) prior to the commencement of the trial when all rats were fed a similar diet. Rats were kept under standard conditions and health status was assessed daily during the experiment.

The experimental groups consisted of: i. Control group (CTR): standard diet: (1 mg/day/rat vitamin E); ii. VITE diet group: (two times greater than NRC recommendations: 2 mg/day/rat VITE); iii. Sunflower oil group (n-6): (gavaged thorough a small plastic feeding tube with 0.5 ml/day/rat sunflower oil+VITE diet); iv. Fish oil group (n-3): (gavaged with 0.5 ml /day/rat fish oil+VITE diet) and v. n-3+n-6 group: (gavaged with 0.3 ml fish oil/day/rat+0.2 ml sunflower oil/day/rat+VITE diet). Each group adapted to the oil gavages over the first four days. The standard and VITE diets were obtained from an industrial animal feed company (Javaneh Khorasan Co., Iran). Rats in all groups were gavaged daily with water in the CTR and VITE groups or oil (n-6, n-3 and their combination). Each rat received 300 mg of C18:2/day, 150 mg of DHA+EPA/day and 100 mg of C18:2 n-6+100 mg of DHA+EPA/day in n-6, n-3 and n-6+n-3 groups, respectively.

It has been suggested that the minimum omega-6 requirement is greater than 1200 mg/100 g of diet (14). In the current study, the rats’ feed intake was weighed (20 g/day/rat), so the minimum requirements were 240 mg linoleic acid (C18:2)/ day/rat. Each rat was gavaged 0.5 ml of sunflower oil received an estimated 300 mg of linoleic acid /day. Fish oil capsules (Nutravite, Canada) were used as the omega-3 source. According to the producer manual, each capsule contained 1000 mg of fish oil with 180 mg of EPA (C20:5 n-3) and 120 mg DHA (C22:6 n-3). Each rat in the n-3 group received 150 mg of DHA+EPA and rats in the n-3+n-6 group received 90 mg of DHA+EPA. The basic CTR and VITE diet contained soybean oil, therefore the omega-6: omega-3 ratio was approximately standard (2-5:1) for all groups. All rats had free access to water.

The body weight of each rat was recorded at the beginning of the study and at the end of follow- up 60 days later. Each cage had 3-4 rats and feed intake was recorded daily for each cage. All major ingredients (soybean, corn) in the standard and VITE diet were the same. Chemical analyses of the experimental diets and the FA compositions of the sunflower oil used in this study are presented in Tables 1 and 2.

All animals were euthanized with ketamine–xylazine10% (100 mg/Kg body weight) after eight weeks and the body weight of each rat was record- ed immediately after euthanization.

**Table 1:** Nutrient analysis of basal diets


Nutrient	Percent

Crude protein	20
Energy	2650 Kcal/ Kg
Crude fat	5
Crude fiber	7.5
Methionine	0.05
Lysine	0.12
Salt	0.05
Ca: P	2


**Table 2:** Fatty acid (FA) composition of sunflower oil used as the n-6 source (percent in total FA)


Fatty acids	Percent

C10:0	-
C12:0	0.1
C12:1	-
C14:0	0.1
C14:1	-
C15:0	-
C15:1	-
C16:0	8
C16:1 trans	0.1
C16:1 cis	0.2
C18:0	3.8
C18:1	28
C18:2 trans	0.3
C18:2	58
C18:3	0.6
C20:0	0.4
C20:3	-
C20:5	-
C20:1	0.2
C22:0	0.5
C22:6	-


### Liver and testes sampling

The liver and right and left testes of each rat were removed, washed in normal saline and immediately weighed as fresh tissue. 

### Semen analysis

The cauda epididymis of the left testis was dissected and placed in 1 ml of pre-warmed tissue culture medium (Medium 199, Sigma, Louis, USA) that contained bovine serum albumin (BSA, Sigma, Louis, USA). Gentle agitation along with tearing of the tissue was applied to enable the spermatozoa to swim out into the medium. The semen samples were incubated at 37˚C for 30 minutes before sperm parammeter analyses were performed. Sperm counts and motility were analyzed by CASA as described previously. The CASA system consists of a phase contrast microscope (Eclipse E-200, Nikon Co., Japan) with a heat plate equipped with Sperm Class Analyzer® software (SCA, full research version 5.1, Microptic Co., Spain). Images are captured by a video camera (Basler Vision, A312FC at 50 fps, Tecnologie Co., Germany) at ×4 magnifications. For this purpose, 5 µl of sperm samples were placed in a Makler chamber. Several fields of view were captured and at least 600 spermatozoa were counted in each analysis (Table 3) (15).

**Table 3:** Standard terminology for variables measured by computer-assisted sperm analyzer (CASA) systems


Parameter	Unit	Description

Curvilinear velocity	µm/seconds	Time-averaged velocity of a sperm head along its actual curvilinear path.
Average path velocity	µm/seconds	Time-averaged velocity of a sperm head along its average path.
Straight-line velocity	µm/seconds	Time-averaged velocity of a sperm head along the straight line between its first detected position and its last.
Amplitude of lateral head displacement	µm	Magnitude of lateral displacement of a sperm head about its average path.
Linearity	%	The linearity of a curvilinear path, VSL/VCL
Wobble	%	A measure of oscillation of the actual path about the average path, VAP/VCL.
Straightness	%	Linearity of the average path, VSL/VAP.
Beat-cross frequency	Hz	The average rate at which the curvilinear path crosses the average path.


VSL; Straight-line velocity, VCL; Curvilinear velocity and VAP; Average path velocity.

### Blood samples and lipid profiles

Blood samples were obtained from rats’ hearts after euthanization and centrifuged at 3000 rpm for 12 minutes at 5˚C to collect the serum. Samples were stored at −20˚C until analysis. Triglyceride (TG), toc tal cholesterol, high density lipoprotein (HDL) and low density lipoprotein (LDL) serum concentrations for rats in all experimental groups were measured by enzymatic colorimetric methods using commercially available kits (Pars Azmoon Laboratories kits, Iran). 

### Statistical analysis

Data were analyzed using SPSS 16 and presented as mean ± SE. ANOVA was used for comparison of means and we considered the confidence level of P<0.05 as significant. The correlation between sperm parameters and blood lipid profiles was evaluated by the correlation bivariate procedure. 

## Results

Feed intake in both the sunflower oil treatments decreased. The lowest (P<0.05) feed intake was demonstrated with the omega-6 source relative to CTR, VITE and fish oil. Body weight did not differ between the five groups during the experiment. Liver and testes weights as well as liver and testes index [organ weight (g)/body weight (g)] were similar among all the treatment groups (Table 4). All organs were of normal appearance.

The effects of the experimental diet and oil source on blood lipid profiles are shown in Table 5. Fish oil and VITE maintained the cholesterol and TG concentrations when compared with the CTR group. The n-6 group had the highest cholesterol, TG and HDL concentrations (P<0.05). Interestingly, n-6+n-3 group had increased LDL concentrations (30 mg/dl) compared with the solely VITE group (17 mg/dl, P<0.05).

**Table 4:** Effects of different treatments on feed intake, body and organ weights of male rats (mean ± SE)


	Treatments				
	CTR	VITE	n-6	n-3	n-6+n-3

Feed intake (g/d)	20 ± 0.1^a^	19.4 ± 0.36^a^	18.2 ± 0.26^b^	19.8 ± 0.19^a^	17.9 ± 0.2^b^
Initial body weight (g)	196 ± 8.9	197 ± 10.5	190 ± 4.1	197 ± 8.1	186 ± 6.4
Final body weight (g)	320 ± 10	326 ± 5	315 ± 8.2	330 ± 7.9	304 ± 8.4
Liver weight (g)	10.3 ± 0.45	10.1 ± 0.69	10.2 ± 0.38	10.7 ± 0.34	9.8 ± 0.31
Right testis (g)	1.4 ± 0.05	1.47 ± 0.07	1.44 ± 0.11	1.55 ± 0.07	1.48 ± 0.09
Left testis (g)	1.48 ± 0.04	1.48 ± 0.07	1.49 ± 0.1	1.54 ± 0.1	1.6 ± 0.08
Liver index	3.23 ± 0.09	3.09 ± 0.18	3.26 ± 0.06	3.25 ± 0.04	3.14 ± 0.11
Right testis index	0.44 ± 0.01	0.45 ± 0.02	0.45 ± 0.02	0.46 ± 0.01	0.48 ± 0.02
Left testis index	0.46 ± 0.01^ab^	0.45 ± 0.02^b^	0.47 ± 0.02^ab^	0.46 ± 0.02^ab^	0.52 ± 0.02^a^


CTR; Standard diet (control), VITE; Diet supplemented with vitamin E, n-6; Gavages sunflower oil and fed VITE diet, n-3; Gavages fish oil and fed VITE diet, n-6+n-3; Gavages sunflower oil with fish oil and fed VITE diet, ^a^ and ^b^; Values with different letters within the same rows are significantly different (P<0.05).

**Table 5:** Effects of different treatments on blood lipid profiles of male rats (mean ± SE)


	Treatments				
	CTR	VITE	n-6	n-3	n-6+n-3

Cholesterol (mg/dl)	58.6 ± 4.1^b^	65.6 ± 2.7^ab^	72.8 ± 3.2^a^	55.4 ± 2.7^b^	69 ± 3.5^a^
Triglyceride (mg/dl)	76.4 ± 9.5^c^	112 ± 11.5^ab^	118 ± 7.6^a^	74 ± 11.5^c^	83 ± 10.2^bc^
HDL (mg/dl)	20.2 ± 1.71^bc^	25.6 ± 1.02^ab^	26.8 ± 1.4^a^	17.2 ± 1.15^c^	22.6 ± 2.8^abc^
LDL (mg/dl)	23.2 ± 1.9^ab^	17.6 ± 2.5^b^	22.4 ±1.9^ab^	24 ± 4.08^ab^	29.8 ± 1.6^a^
LDL: HDL	1.16 ± 0.11^ab^	0.7 ± 0.11^b^	0.83 ± 0.04^b^	1.4 ± 0.23^a^	1.43 ± 0.23^a^


CTR; Standard diet (control), VITE; Diet supplemented with vitamin E, n-6; Gavages sunflower oil and fed VITE die, n-3; Gavages fish oil and fed VITE diet, n-6+n-3; Gavages sunflower oil with fish oil and fed VITE diet, ^a, b^and ^c^; Values with different letters within the same rows are significantly different (P<0.05).

Sperm quality and quantity were significantly affected by the treatments (Table 6). Fish oil and VITE supplementation showed a positive effect on sperm concentration (73×10^6^) compared with the CTR (53×10^6^) and n-3+n-6 group (65.8×10^6^) (P<0.05). Sperm viability improved in the VITE group compared to the CTR group. The percent of sperm with progressive movement increased in the VITE and fish oil groups, with the highest percentage of progressive sperm observed in the VITE group compared with the CTR group (P<0.05). However, fish oil did not improve this parameter dramatically. For all of the above parameters, gavages of sunflower oil alone or in combination with fish oil showed moderate effect. Of note, the VITE group had a reduced number of non-progressive sperm (19%) compared to the CTR group (27%, P<0.05). However, there was little effect on the number of non-progressive sperm in the oil groups. Rats from the n-3 group had 16% immotile sperm, however there were 31% observed in the CTR group (P<0.05). Overall for sperm kinematic parameters, VITE significantly improved motion patterns which it is constant by fish oil. Nevertheless oil consumption cannot improved some parameters, linearity (LIN), straightness (STR) and wobble (WOB) of sperm were the highest in the VITE groups compared with the CTR group (P<0.05) (Table 6). In all experimental rats, only cholesterol had a significant negative correlation with the percentage of sperm with STR parameter (r=-0.40, P=0.047).

**Table 6:** Effects of different treatments on sperm parameters (mean ± SE)


	Treatments				
	CTR	VITE	n-6	n-3	n-6+n-3

Concentration (×10^6^)	53.4 ± 2.72^c^	69 ± 1^ab^	69.1 ± 2.4^ab^	73.1 ± 1.7^a^	65.8 ± 3.2^b^
Viability (%)	69 ± 2.3^b^	81 ± 4.7^a^	78 ± 2.3^ab^	84 ± 1.3^a^	77 ± 3.7^ab^
Progressive motility (%)	42 ± 4.8^b^	62 ± 6.3^a^	55 ± 3.6^ab^	63 ± 3.7^a^	56 ± 5.2^ab^
Non-progressive motility (%)	27 ± 3.1^a^	19 ± 2.18^b^	22 ± 1.6^ab^	21 ± 2.7^ab^	21 ± 1.6^ab^
Immotile (%)	31 ± 2.3^a^	19 ± 4.6^b^	23 ± 2.1^ab^	16 ± 1.1^b^	23 ± 3.5^ab^
VCL (µm/s)	64.6 ± 8.5	80 ± 5.2	74 ± 2.8	81 ± 4.6	81 ± 4.1
VSL (µm/s)	13.7 ± 1.8^c^	19 ± 0.8^ab^	14.7 ± 0.6^bc^	21 ± 1.8^a^	18.4 ± 1.6^ab^
VAP (µm/s)	26 ± 3.7^c^	38 ± 1.8^ab^	31.5 ± 1^bc^	41 ± 2.4^a^	34 ± 2.8^ab^
LIN (%)	18.8 ± 1.4^d^	23 ± 0.91^ab^	20 ± 0.52^cd^	26 ± 0.8^a^	22 ± 1.1^bc^
STR (%)	45 ± 2.3^bc^	50 ± 0.47^ab^	45.5 ± 0.6^bc^	51 ± 1.06^a^	44 ± 1.7^c^
WOB (%)	40 ± 1.3^d^	48 ± 1.4^ab^	42.5 ± 0.3^cd^	51 ± 0.92^a^	45 ± 1.4^bc^
ALH (Hz)	2.07 ± 0.15	2.30 ± 0.12	2.26 ± 0.085	2.3 ± 0.085	2.4 ± 0.125
BCF (µm)	6.2 ± 0.4^c^	7.7 ± 0.33^ab^	6.8 ± 0.16^bc^	8.11 ± 0.20^a^	7.6 ± 0.4^ab^

CTR; Standard diet (control), VITE; Diet supplemented with vitamin E, n-6; Gavages sunflower oil and fed VITE diet, n-3; Gavages fish oil and fed VITE diet, n-6+n-3; Gavages sunflower oil with fish oil and fed VITE diet, ^a, b , c, d^; Values with different letters within the same rows are significantly different (P<0.05), VCL; Curvilinear velocity, VSL; Straight-line velocity, VAP; Average path velocity, LIN; Linearity, STR; Straightness, WOB; Wobble, ALH; Amplitude of lateral head displacement and BCF; Beat-cross frequency.

## Discussion

The significant decrease in feed intake with sunflower oil use suggested that omega-6 FA had an impact on short-term feed intake regulation. Greenberg et al. (16) similarly demonstrated that infusion of fat into the rat intestine decreased food intake. It appeared that the potential of the omega-6 source to depress feed intake was stronger than the omega-3 source. In dairy cattle, the decrease in feed intake during an infusion of sunflower oil in the abomasum that contained no detectable linolenic acid (C18:3) was greater than either canola or soybean oil which contained 7% linolenic acid. Nevertheless, Yan et al. (10) did not report any significant difference in the effect on feed intakes of several dietary omega-3: omega-6 ratios, but rats on a diet with the highest omega-3: omega 6 diet ratio had the highest intake. Our data provide evidence that omega-6 FA may decrease feed intake in rats despite presence of VITE. However, liver and testis weights were maintained and organ indices were consistent with an unaltered body weight. Yan et al. (10) alongside our results reported that feeding diets which contained several omega-6: omega-3 ratios provided by soybean/flaxseed oil had no effect on testis index. 

Although rat sperm has been shown to contain high levels of omega-6 FA (17); dietary VITE dramatically improved sperm quality. The positive effects of dietary omega-3 FA from fish oil on sperm quality have been shown in a number of species such as humans and ruminants (7,9,18,19) for which the sperm contain high levels of C22:6 n-3 (DHA). Interestingly, in current study and in species where n-6 FAs are predominant in sperm, it has been shown that dietary fish oil can elevate both sperm quality and quantity in rabbits (20), horses (21) and boars (22) as well as in domestic birds such as roosters and turkeys (23). Similarly, Yan et al. (10) have suggested that omega-3 FA obtained from plant sources improved rat sperm parameters. Omega-6 and omega-3 PUFAs are incorporated into spermatozoa cell membranes and omega-3 PUFAs improve semen quality via antioxidant activity (24). 

It appears that improved sperm motility was the result of the antioxidant effects of omega-3 FA or VITE. In the current study, sperm motivation parameters were elevated by dietary VITE, especially by fish oil+VITE consumption. A positive relation between dietary fish oil supplementation and number of motile spermatozoa has been proposed (9); however, all the rams in this study received VITE supplementation. 

The tail of the sperm is mostly associated with sperm movement, whereas the function of the head is associated with the acrosome reaction and membrane fusion (25). Thus, a putative mechanism is that VITE consumption increases the preservation and presence of DHA in the sperm tail which might improve motility. Additionally, VITE or DHA in spermatozoa might have specific functions unrelated to fluidity, which are similar to their functions in the brain and retina (26). Altogether our findings have shown that dietary VITE with or without omega-3 can improve sperm motility parameters if consumed for eight weeks in rat. Meanwhile, previous studies assumed that all improvements of sperm parameters are related to the consumption of dietary fatty acids, especially omega-3. 

The role of VITE as an antioxidant has been proven, but the effect of dietary VITE on semen and sperm is controversial. Moreover, in several previous studies, a negative effect of PUFA consumption in the absence of antioxidant was reported (6,10). For this reason, we expected that dietary omega-3 and omega-6 fatty acid without antioxidant could destroy spermatogenesis, a hypothesis supported by our own unpublished data in rams, especially in frozen-thawed semen. What is new in the current study is the emphasis on the antioxidant-PUFA combination. In roosters normal semen are unresponsive to dietary increases in VITE (5), however Gökçen et al. (27) have reported an enhancement in the motility of ram sperm with dietary VITE supplementation. In previous studies, unsaturated FA supplementation has been shown to disturb sperm parameters in the wild rodent, *Calomys laucha* (12), or ram (28) when used without VITE supplementation. Similarly, in a study by Yan et al. (10), there was a significant reduction of motile sperm in the group which consumed the highest level of omega-3 FA compared with the recommended ratio contributed to decreased dietary antioxidant support. The observed reduction in the percent of non-progressive sperm in the VITE diet compared with the CTR group while feeding oil caused intermediate effects was one of the novel findings of the present study. Possibly dietary VITE could decrease the percent of non-progressive sperm while dietary FA could change motility another way, such as decreasing the numbers of immotile sperm. This finding could be a focus for treatment strategies or improvements in sperm motility in subfertile or infertile humans and animals. Dietary VITE supplementation may manipulate FA profiles of sperm which improve sperm parameters. 

The positive effects of VITE and omega-3 fatty acids were clearer when sperm kinematic parameters were investigated. Increased straight-line velocity (VSL) and average path velocity (VAP) by increased dietary omega-3 concentration were consistent with motility data. Feeding omega-3 FA from fish or flaxseed oil compared with corn oil has been shown to affect sperm kinematic parameters in roosters (23), most likely because sperm cell membrane fluidity and flexibility are increased by the consumption of omega-3 FA and VITE. It is surprising to note that rooster sperm contain as high a level of omega-6 FA as the rats in the current study (3). 

In previous studies, our team demonstrated that fish oil could improve testosterone concentration (9) and decrease ROS (6). Although dietary use of lipid supplements normally increases blood cholesterol (29) as shown in the n-6 or n-6+n-3 groups, the use of omega-3 FA reduced cholesterol as well as TG levels. Our data, together with findings from previous studies demonstrated the cholesterol-lowering effects of n-3 FA. The decrease in cholesterol and TG concentration suggests that omega-3 FA results in an altered turnover of hepato-peripheral lipids and has mild lipotropic effects compared with omega-6. Fish oils are believed to primarily reduce TG levels by promoting FA degradation via paroxysmal β-oxidation, inhibit lipogenesis in the liver, and accelerate the clearance of plasma TG (30). 

Significant change in LDL levels was observed in rats fed supplemental fish oil. Popović et al. (31) 

demonstrated a significant decrease in plasma TG and LDL concentration, and increase in the level of HDL in rats fed omega-3 FA (31). However, the beneficial effects of fish oil on LDL and/or HDL appear inconsistent, whereas influences of particle size seem to be key (32). 

There was no strong correlation between lipid profiles and sperm parameters in the current study, especially in the FA group. The oxidative process in serum and tissues, particularly the oxidative conversion of native LDL to oxidized LDL by free radicals, is now considered to be associated with male infertility (33). The positive relation between LDL and sperm linearity motility could be due to increased dietary supply of antioxidants and the prevention of oxidized LDL’s negative effect in the testes. Improved sperm motility and viability by the VITE diet support the above reasoning. 

## Conclusion

The effectiveness of dietary vitamin E on sperm was proven by enhanced sperm kinematics parameters. Omega-6 FA reduced short-term feed intake and it appeared that in a diet supplemented by vitamin E the depression potential of omega-6 on food intake was stronger than omega-3 in mature male rats. There were no strong correlations between sperm parameters and blood lipid profiles. Although fatty acids play a pivotal role in sperm, our findings supported the unique view that sperm quality in fertile male rats can be improved by VITE supplementation alone. 

## References

[1] Danikowski S, Sallmann HP, Halle I, Flachowsky G (2002). Influence of high levels of vitamin E on semen parameters of cocks. J Anim Physiol Anim Nutr (Berl).

[2] Kawano N, Yoshida K, Miyado K, Yoshida M (2011). Lipid rafts: keys to sperm maturation, fertilization, and early embryogenesis. J Lipids.

[3] Speake BK SP, Rooke JA, De Vriese SR, Christophe AB (2003). Regulation of avian and mammalian sperm production by dietary fatty acids. Male fertility and lipid metabolism.

[4] Surai PF, Kutz E, Wishart GJ, Noble RC, Speake BK (1997). The relationship between the dietary provision of α-tocopherol and the concentration of this vitamin in the semen of chicken: effects on lipid composition and susceptibility to peroxidation. J Reprod Fertil.

[5] Alizadeh AR, Alikhani M, Ghorbani GR, Rahmani HR, Rashidi L, Loor JJ (2012). Effects of feeding roasted safflower seeds (variety IL-111) and fish oil on dry matter intake, performance and milk fatty acid profiles in dairy cattle. J Anim Physiol Anim Nutr(Berl).

[6] Esmaeili V, Shahverdi AH, Moghadasian MH, Alizadeh AR (2015). Dietary fatty acids affect semen quality: a review. Andrology.

[7] Gholami H, Chamani M, Towhidi A, Fazeli MH (2010). Effect of feeding a docosahexaenoic acid-enriched nutriceutical on the quality of fresh and frozen-thawed semen in Holstein bulls. Theriogenology.

[8] Safarinejad MR (2011). Effect of omega-3 polyunsaturated fatty acid supplementation on semen profile and enzymatic anti-oxidant capacity of seminal plasma in infertile men with idiopathic oligoasthenoteratospermia: a double-blind, placebo-controlled, randomised study. Andrologia.

[9] Esmaeili V, Shahverdi AH, Alizadeh AR, Alipour H, Chehrazi M (2014). Saturated, omega-6 and omega-3 dietary fatty acid effects on the characteristics of fresh, frozen-thawed semen and blood parameters in rams. Andrologia.

[10] Yan L, Bai XL, Fang ZF, Che LQ, Xu SY, Wu D (2013). Effect of different dietary omega-3/omega-6 fatty acid ratios on reproduction in male rats. Lipids Health Dis.

[11] Castellano CA, Audet I, Bailey JL, Chouinard PY, Laforest JP, Matte JJ (2010). Effect of dietary n-3 fatty acids (fish oils) on boar reproduction and semen quality. J Anim Sci.

[12] Cardoso TF, Varela AS Jr, Silva EF, Vilela J, Hartmann A, Jardim RD (2014). Influence of mineral, olive or sunflower oils on male reproductive parameters in vitro the wild rodent Calomys laucha. Andrologia.

[13] Blasbalg TL, Hibbeln JR, Ramsden CE, Majchrzak SF, Rawlings RR (2011). Changes in consumption of omega-3 and omega-6 fatty acids in the United States during the 20th century. Am J Clin Nutr.

[14] Bourre JM, Piciotti M, Dumont O, Pascal G, Durand G (1990). Dietary linoleic acid and polyunsaturated fatty acids in rat brain and other organs.Minimal requirements of linoleic acid. Lipids.

[15] van der Horst G, Maree L, Kotzé SH, O’Riain MJ (2011). Sperm structure and motility in the eusocial naked mole-rat, Heterocephalus glaber: a case of degenerative orthogenesis in the absence of sperm competition?. BMC Evol Biol.

[16] Greenberg D, Smith GP, Gibbs J (1990). Intraduodenal infusions of fats elicit satiety in sham-feeding rats. Am J Physiol.

[17] Hall JC, Hadley J, Doman T (1991). Correlation between changes in rat sperm membrane lipids, protein, and the membrane physical state during epididymal maturation. J Androl.

[18] Samadian F, Towhidi A, Rezayazdi K, Bahreini M (2010). Effects of dietary n-3 fatty acids on characteristics and lipid composition of ovine sperm. Animal.

[19] Giahi L, Mohammadmoradi S, Javidan A, Sadeghi MR (2016). Nutritional modifications in male infertility: a systematic review covering 2 decades. Nutr Rev.

[20] Mourvaki E, Cardinali R, Dal Bosco A, Corazzi L, Castellini C (2010). Effects of flaxseed dietary supplementation on sperm quality and on lipid composition of sperm subfractions and prostatic granules in rabbit. Theriogenology.

[21] Brinsko SP, Varner DD, Love CC, Blanchard TL, Day BC, Wilson ME (2005). Effect of feeding a DHA-enriched nutriceutical on the quality of fresh, cooled and frozen stallion semen. Theriogenology.

[22] Rooke JA, Shao CC, Speake BK (2001). Effects of feeding tuna oil on the lipid composition of pig spermatozoa and in vitro characteristics of semen. Reproduction.

[23] Bongalhardo DC, Leeson S, Buhr MM (2009). Dietary lipids differentially affect membranes from different areas of rooster sperm. Poult Sci.

[24] Safarinejad MR, Safarinejad S (2012). The roles of omega-3 and omega-6 fatty acids in idiopathic male infertility. Asian J Androl.

[25] Argov-Argaman N, Mahgrefthe K, Zeron Y, Roth Z (2013). Variation in lipid profiles within semen compartment--the bovine model of aging. Theriogenology.

[26] Zalata AA, Christophe AB, Depuydt CE, Schoonjans F, Comhaire FH (1998). The fatty acid composition of phospholipids of spermatozoa from infertile patients. Mol Hum Reprod.

[27] Gökçen H, Çamaş H, Erdinģ H, Aşti R, Çekgül E, Şener E (1990). Studies on the effects of vitamin E and selenium added to the ration on acrosome morphology, enzyme activity and fertility of frozen ram spermatozoa. Turk J Vet Anim Sci.

[28] de Graaf SP, Peake K, Maxwell WMC, O'Brien JK, Evans G (2007). Enfluence of supplementing diet with Oleic and Linoleic acid on the freezing ability and sex-sorting parameters of ram semen. Livestock Science.

[29] Harland JI (2012). Food combinations for cholesterol lowering. Nutr Res Rev.

[30] Jacobson TA, Glickstein SB, Rowe JD, Soni PN (2012). Effects of eicosapentaenoic acid and docosahexaenoic acid on lowdensity lipoprotein cholesterol and other lipids: a review. J Clin Lipidol.

[31] Popović T, Borozan S, Arsić A, Martačić JD, Vučić V, Trbović A (2012). Fish oil supplementation improved liver phospholipids fatty acid composition and parameters of oxidative stress in male wistar rats. J Anim Physiol Anim Nutr(Berl).

[32] Riediger ND, Othman RA, Suh M, Moghadasian MH (2009). A systemic review of the roles of n-3 fatty acids in health and disease. J Am Diet Assoc.

[33] Kosola J, Vaara JP, Ahotupa M, Kyrolainen H, Santtila M, Oksala N (2013). Elevated concentration of oxidized LDL together with poor cardiorespiratory and abdominal muscle fitness predicts metabolic syndrome in young men. Metab Clin Exp.

